# Butanol production from lignocellulosic biomass: revisiting fermentation performance indicators with exploratory data analysis

**DOI:** 10.1186/s13068-019-1508-6

**Published:** 2019-06-28

**Authors:** Cansu Birgen, Peter Dürre, Heinz A. Preisig, Alexander Wentzel

**Affiliations:** 1 Department of Chemical Engineering, NTNU, 7491 Trondheim, Norway; 2 Institute of Microbiology and Biotechnology, Ulm University, 89069 Ulm, Germany; 3 SINTEF Industry, 7465 Trondheim, Norway

**Keywords:** Butanol, *Clostridia*, ABE fermentation, Lignocellulosic biomass, Mixed sugars, Exploratory data analysis

## Abstract

After just more than 100 years of history of industrial acetone–butanol–ethanol (ABE) fermentation, patented by Weizmann in the UK in 1915, butanol is again today considered a promising biofuel alternative based on several advantages compared to the more established biofuels ethanol and methanol. Large-scale fermentative production of butanol, however, still suffers from high substrate cost and low product titers and selectivity. There have been great advances the last decades to tackle these problems. However, understanding the fermentation process variables and their interconnectedness with a holistic view of the current scientific state-of-the-art is lacking to a great extent. To illustrate the benefits of such a comprehensive approach, we have developed a dataset by collecting data from 175 fermentations of lignocellulosic biomass and mixed sugars to produce butanol that reported during the past three decades of scientific literature and performed an exploratory data analysis to map current trends and bottlenecks. This review presents the results of this exploratory data analysis as well as main features of fermentative butanol production from lignocellulosic biomass with a focus on performance indicators as a useful tool to guide further research and development in the field towards more profitable butanol manufacturing for biofuel applications in the future.

**Electronic supplementary material:**

The online version of this article (10.1186/s13068-019-1508-6) contains supplementary material, which is available to authorized users.

## Introduction

Chemicals and fuels from renewable resources have gained global interest due to rising global warming and climate change concerns, volatility of oil price and supply, and legal restrictions on nonrenewable energy sources [1]. *n*-Butanol (in the further run for simplicity reasons referred to as butanol) is a promising biofuel alternative based on several advantages compared to the more established biofuels ethanol and methanol: a longer carbon chain length and thus a higher heating value, as well as lower volatility, polarity, corrosivity and heat of vaporization, leading to lesser ignition problems. Moreover, diesel engines can run on pure butanol or diesel blends without any modifications and apparent damage [2]. A more detailed overview of the physical properties of butanol in comparison to other biofuels, gasoline and diesel can be found elsewhere in literature [3, 4].

### A brief history of biological butanol production

Biological production of butanol under anaerobic conditions is typically referred to in literature as a part of ‘ABE fermentation’, since acetone, butanol and ethanol are usually produced simultaneously in this process. Louis Pasteur was the first to report about microbial butanol production in 1862 [5]. However, the first production utilizing the Weizmann process began only in 1913, aiming to produce acetone for rubber synthesis [6]. Later in 1916, the first industrial-scale ABE fermentation began operation due to a high demand for acetone during World War I, and after the armistice in November 1918, most of the plants were shut down [7].

Industrial ABE fermentation, however, kept expanding worldwide, facilitated by the usability of butanol as a solvent [6]. In 1945, two-thirds of the butanol and one-tenth of the acetone in the U.S. were produced by ABE fermentation processes. However, their share in the total output declined rapidly during the 1950s mainly because of the acute competition with the expanding petrochemical industry and decreasing feedstock availability [8]. ABE fermentation became popular again in the 1970s after the oil crisis, and it has since been gaining increasing interest owing to the advancements in Metabolic Flux Analysis (1984), Metabolic Engineering (1992), Gene KO Homologous Recombination (1994), and Complete Genome Sequencing (2001) [7], holding promise of improved production yields and productivities for more economic microbial production processes. There are several excellent reviews covering the historical development of ABE fermentation in detail [6, 7, 9–11].

### Previous reviews covering the topic

There are several challenges such as high substrate cost, solvent toxicity, low cell density and by-product formation that need to be addressed for sustainable and economical fermentative butanol production. These issues cause low butanol yield, titer, productivity and selectivity.

Great efforts have been made to find cheap/free feedstock and cost efficient processing methods to overcome the high substrate cost problem, and several review papers address this issue in detail [3, 12–18]. Low solvent tolerance limits the butanol titer to maximum 2% dependent of the strain used [19], causing high downstream processing cost; therefore some reviews collected and discuss information on this specific challenge [20, 21]. Efficient separation of butanol from the fermentation mixture is another important topic with several reviews discussing particularly downstream processes for ABE fermentation [3, 22]. Strain improvement by metabolic engineering has an important role in optimizing butanol production. For details, readers can refer to “Strain development” section below, as well as the review papers published on the features of clostridial pathways and metabolic engineering of butanol producers [23–31].

Main issues and possible solutions discussed in previous review papers are summarized in Table 1 that provides a comprehensive overview in terms of their frequency of appearance.

**Table 1:** Summary of main challenges and solutions for fermentative butanol production

Challenge	Suggested solution
High substrate cost	Lignocellulosic substrates [3, 12–15, 18, 23, 29, 31–35] Starch based waste [12, 29, 33] Syngas [12, 23, 24, 33, 35] Macroalgae [12, 16, 23] Crude glycerol [12, 23, 24, 31] Protein waste [23] Whey permeate [14, 29, 34] Economical feedstock processing methods [3, 18, 29] Medium optimization [18, 28] Inulin [31]
Low butanol selectivity	Metabolic engineering for disruption of the pathway for acetone [3, 13–15, 23, 25, 27, 32, 34] Homo-butanol fermentation via chemical mutagenesis and metabolic engineering [23, 24, 33, 35] Conversion of acetone into isopropanol [13, 15, 23] Decoupling sporulation from solventogenesis [3, 13, 14, 23, 25, 27, 28, 34, 35]
Low butanol titer	Metabolic engineering and mutagenesis for higher butanol tolerance [13–15, 21, 23–25, 27, 28, 32–35] In situ product removal [3, 12–15, 18, 22, 23, 27, 28, 32, 34, 35] Introducing butanol pathways in other hosts [3, 13, 15, 21, 23–25, 27, 33–35] Re-enforcing hot channel for butanol formation [14]
Low butanol yield	Simultaneous utilization of mixed sugars in the hydrolysate without *Carbon Catabolite Repression* [14, 23, 31] Extending the substrate utilization range [15, 34, 35]
Low butanol productivity	Simultaneous utilization of mixed sugars without *Carbon Catabolite Repression* [3, 23, 28, 29] Fed-batch fermentation [3, 12, 14, 18, 34] Chemostat/continuous culturing [3, 12–15, 18, 32, 34] Immobilized cell chemostat [3, 12–15, 18, 34] Cell recycle chemostat [3, 12–15, 18, 34] Multi stage chemostat [3, 13, 14, 18]
Low O_2_ tolerance	Co-culturing to maintain anaerobic conditions [32] Random mutagenesis and selection [13, 35] Metabolic engineering [27, 36]
Culture degeneration	Prevention of excessive acidification of the culture [35]
Phage contamination	Good factory hygiene, strains immune to specific phages [27, 35]

### The aim and scope of the present review

The aim of the present review is to provide a critical overview of existing literature on fermentative butanol production from lignocellulosic biomass and mixed sugars in batch mode with a focus on performance indicators. A comprehensive collection of data derived from original literature of the last 3 decades thereby laid the basis for performing exploratory data analysis (EDA).

## Fermentative butanol production from lignocellulosic biomass

**Fig. 1:**
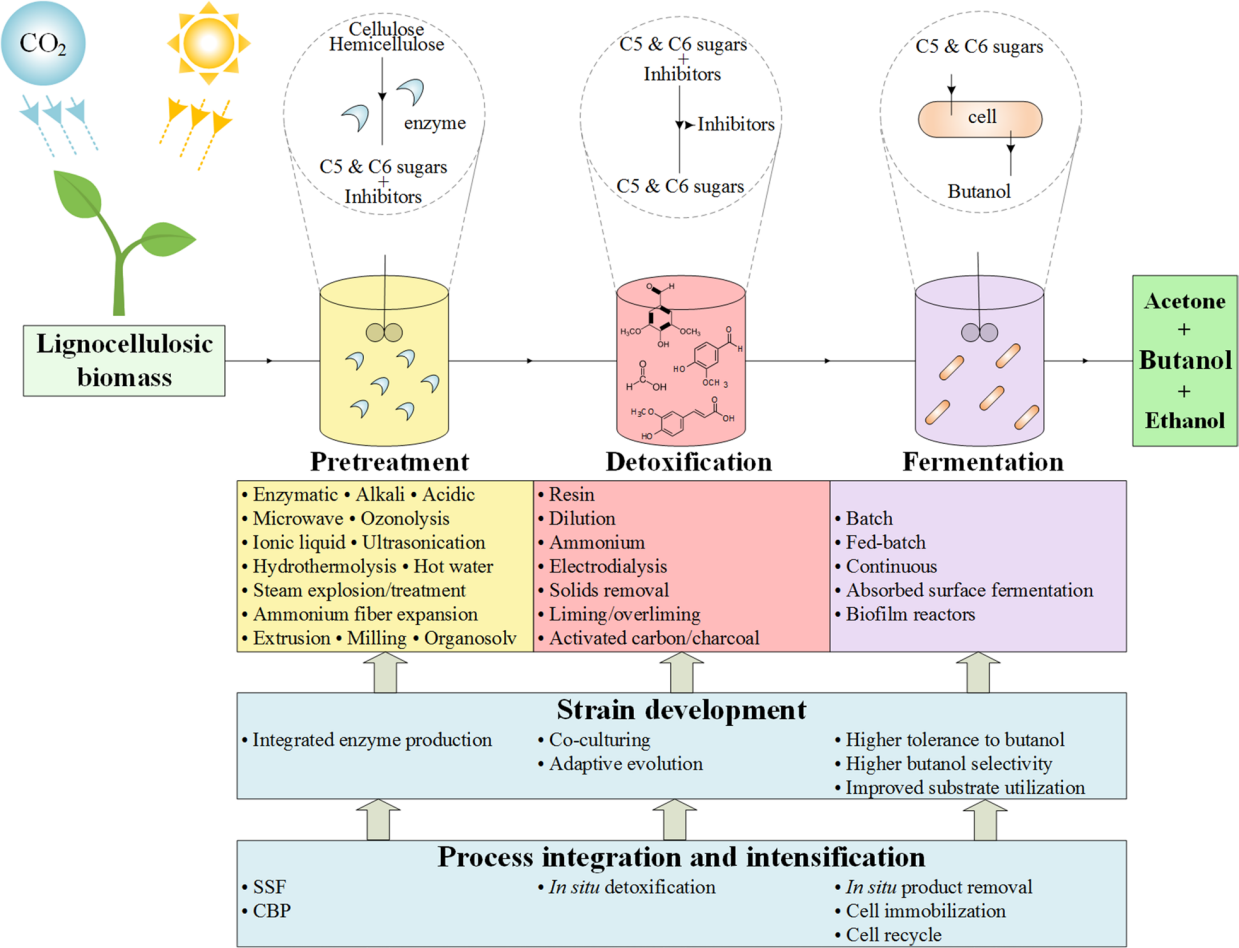
A representative schematic diagram of fermentative butanol production from lignocellulosic biomass

A typical conversion process from lignocellulosic biomass to butanol involves three major steps: pretreatment, detoxification and fermentation. A representative schematic diagram of the process is shown in Fig. 1.

### Pretreatment

**Fig. 2:**
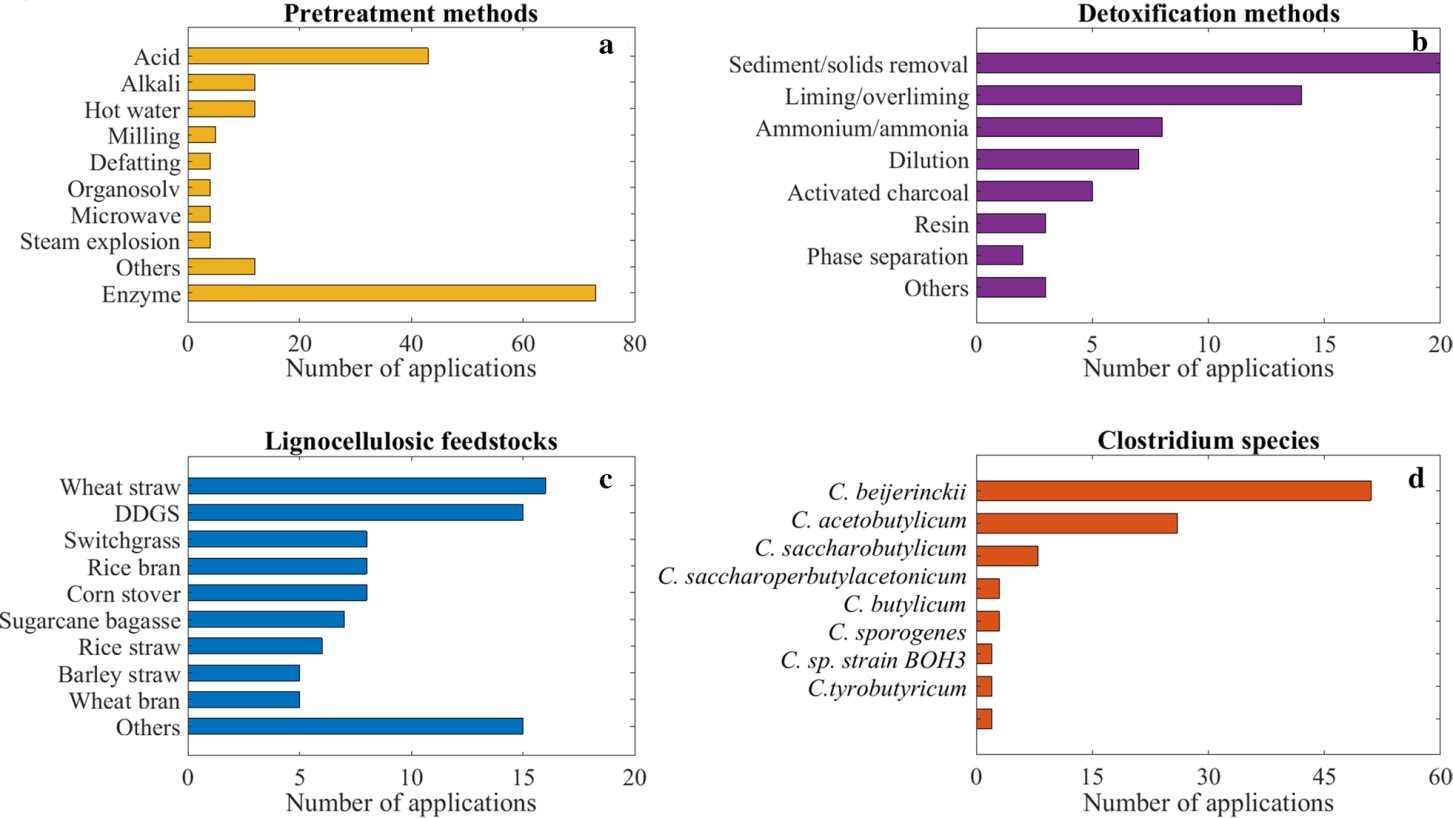
Common pretreatment methods (**a**), detoxification methods (**b**), lignocellulosic feedstocks (**c**), and Clostridium strains (**d**) used in fermentative butanol production from lignocellulosic biomass

Lignocellulosic biomass is a favorable feedstock since it is the most abundant renewable biomass resource on the planet, and, compared to sugars from e.g. sugar cane or maize, it avoids direct fuel-versus-food competition. It is the feedstock for butanol production suggested most frequently in literature as shown in Table 1. Its main constituents are cellulose, hemicellulose and lignin [37]. The opening of the lignocellulosic biomass structure and the release of sugar content from hemicellulose and cellulose with other cross-linked units and the residual non-hydrolyzed raw feedstock is called pretreatment [38]. Conversion of biomass into its main constituents is referred to in literature as fractionation, which is sometimes used interchangeably with pretreatment, i.e. pretreatment is mentioned as a way of achieving biomass fractionation, or the term fractionation is used as (part of) a pretreatment method [13, 39, 40]. In the present study, for simplicity reasons we name all steps involved in the conversion of the feedstock to sugars as pretreatment though enzymatic hydrolysis of the polysaccharide fractions is often referred to as a step that is distinct from other pretreatment measures. Predominance of enzymatic hydrolysis in the pretreatment methods in Fig. 2a shows its widespread application to produce fermentable sugars from lignocellulosic biomass.

Milling/grinding, extrusion, microwave and ultra-sonication are common physical pretreatment methods that open up the physical structure of lignocellulosic biomass [41–47]. Physico-chemical methods such as steam explosion, steam treatment, hydrothermolysis, ammonium fiber expansion, and hot water treatment cause both the structure to unravel and a release of sugar monomers and dimers [42, 48–52]. Major chemical pretreatment methods are alkali, acidic, ozonolysis, ionic liquid and organosolv treatments [41–44, 48–73].

Enzymatic hydrolysis using suitable enzyme mixtures degrades polysaccharides such as cellulose and xylan to fermentable C6 and C5 sugar monomers, respectively [74]. Typically, combinations of several of the above-mentioned pretreatment methods are employed depending on the feedstock. Operating conditions of pretreatment are crucial since a small change in the operating parameters can cause great differences in reduced sugar composition and concentration as well as inhibitory compounds, consequently negatively affecting enzymatic hydrolysis, fermentability and the cost of substrate [18]. Therefore, it is crucial to examine the feasibility of any pretreatment method with respect to the generation of inhibitors, energy consumption, operating cost, and sugar yield.

### Detoxification

Compounds that are inhibitory to microorganisms and enzymes are often generated during pretreatment [74]. Cellulose and hemicellulose should ideally only yield sugar monomers such as glucose, xylose, and mannose. However, severity of some pretreatment conditions converts those sugars into furfural, 5-hydroxymethyl furfural (HMF), formic acid, acetic acid, levulinic acid and salts, which can be inhibitory [18, 75]. Partial decomposition of lignin generates inhibitory (poly)phenolic aromatic compounds such as *p*-coumaric acid, ferulic acid, syringe aldehyde, vanillic acid and vanillin [18]. Contrary to ethanol-producing microorganisms like the yeast *Saccharomyces cerevisiae*, furfural, HMF or acetic acid are not inhibitory to clostridial butanol producers at relatively low concentrations, rather they are reported to be stimulatory [76]. Another common compound generated during pretreatment of lignocellulosic biomass is formic acid. It is found to be inhibitory to *C. acetobutylicum* at 0.5 g/l [77] and 0.074 g/l (1 mM) inside the cell wall [78] due to acid crash [79]. Therefore, if larger amounts of inhibitors are present after pretreatment, it is a necessity to remove these for a successful fermentation. For this purpose, several detoxification methods such as electrodialysis [66], liming/overliming [48, 49, 57–59, 73], activated carbon/charcoal [50, 51, 71, 72], dilution [44, 66], and resin treatments [54, 63] are applied. Even though it is not specifically mentioned as a detoxification method, solid/sediment removal by filtration or centrifugation is also commonly applied to alleviate the inhibitory effects of the solids and undissolved lignin in the lignocellulosic hydrolysates [41, 53, 54, 59, 63, 66]. It is important to note that the enzymes used in the hydrolysis step can be inhibited by the compounds mentioned above as well as their sugar yields, which can impose a limit to high substrate concentration [74]. Alternative lines of research currently target new pretreatment methods that are less prone to inhibitor formation (like organosolv or other low-temperature methods) and thus ideally do not require detoxification prior to fermentation, as well as increasing the inhibitor tolerance of fermentation strains e.g. by means of adaptive evolution.

### Fermentation

ABE fermentation is biphasic; first, acetic acid and butyric acid are produced in the acidogenesis phase, then the acids are re-assimilated to yield the solvents acetone, butanol and ethanol [80]. Batch fermentation is the most studied mode due to simple operation and low risk of contamination [81], and readers can access numerous original studies of batch fermentations of lignocellulosic biomass to produce butanol [41–44, 48–73]. Low cell density can result in low productivity, and absorbed substrate fermentation [82] and biofilm reactors [83] have been applied to overcome this problem in batch processes. Fed-batch mode is beneficial to tackle substrate inhibition by gradually adding the substrate, thus keeping the substrate concentration below toxic levels [84]. However, fed-batch fermentation should still be accompanied by in situ product removal to alleviate product inhibition [60, 85, 86]. Continuous fermentation (chemostat) has advantages over batch and fed-batch modes such as improved productivity [84]. Multi-stage [87], immobilized cell [88, 89], cell recycling and bleeding [90, 91] techniques have been applied to improve chemostat performance.

### Strain development

Strain development refers to any modifications in the butanol production strain done by random mutagenesis and selection, like in adaptive laboratory evolution, or directed, rational and/or systems biology guided genetic modification employing metabolic engineering and synthetic biology to improve fermentation performance by means of increased tolerance to toxic components, butanol selectivity and productivity, and improved substrate utilization and range.

In general, detoxification methods shown in Fig. 2a are used for removal of inhibitors present in the substrate and/or feedstock as described in “Detoxification” section. Co-culturing with other species to eliminate toxic components such as oxygen in case of anaerobic fermentation is an alternative method [32]. Random mutagenesis and selection [13, 35], and metabolic engineering [27, 36] have been applied for the same purpose. Inhibition due to butanol accumulation is one of the greatest challenges. Therefore, metabolic engineering and mutagenesis have been targeting this specific problem as well by developing strains with greater to resistance to butanol toxicity [13–15, 21, 23–25, 27, 28, 32–35].

A typical fermentative butanol production yields acetone and ethanol as well, which decreases the selectivity of the product of interest. Metabolic engineering for disruption of acetone producing pathways [92], homo-butanol fermentation via chemical mutagenesis and metabolic engineering and conversion of acetone into isopropanol are among the strategies developed to address this issue.

Efficient utilization of the substrate is crucial to achieve a high butanol yield, thus improving fermentation performance [14, 23, 31]. Disrupting the genes responsible for *Carbon Catabolite Repression* and overexpression of genes responsible for xylose transport and catalytic enzymes (d-xylose isomerase, xylulokinase, and enzymes of PPP) are commonly followed approaches [71, 72, 93, 94].

It is important to mention the recent efforts on CRISPR-Cas9 genome engineering systems to improve butanol production by fermentation. Most of the research focuses on production by *Escherichia coli* [95]. However, Clostridial butanol production improvements have been achieved by using this technique as well [96].

In summary, the increasing numbers of publications in recent years employing strain engineering techniques and approaches to address key bottlenecks in clostridial butanol production hold promise to finally solving these in the future.

### Process integration and intensification

Process integration and intensification techniques are applied to obtain cost-effective fermentation processes. Important process intensification approaches include (a) simultaneous saccharification and (co-)fermentation (SSF or SSCF) in which hydrolysis of polysaccharides present in (pre-treated) biomass is performed by externally produced and added hydrolytic enzyme mixes in situ with the simultaneous fermentation of the liberated sugars by a strain (or in the case of SSCF several strains with complementary sugar substrate spectrum) producing the product of choice, e.g. butanol [42, 53], and (b) consolidated bioprocessing (CBP) in which the saccharolytic enzymes are produced within the sugar fermenting culture e.g. by the target product producing strain itself or in co-culture with a partner strain specialized in enzyme production and secretion [97].

Gas stripping, pervaporation, adsorption, liquid–liquid extraction, pertraction (membrane extraction), reverse osmosis and membrane distillation are in situ product removal methods used to alleviate inhibitory effects of butanol [3, 22]. Fermentation with integrated gas stripping has widely been studied, mostly in fed-batch mode, which showed improved butanol productivity [60, 85].

Cell immobilization and cell recycle are mostly integrated to fermenters operated in continuous mode to improve butanol productivity by preventing the loss of cell mass with the bleeding stream out from the fermenter.

Process integration and intensification measures therefore play crucial roles in optimizing butanol fermentation processes for improved performance and economic competitiveness.

## Fermentative butanol production from mixed sugars

There has been a great scientific interest in the utilization of different sugars in mixed form for the production of biofuels, since pre-processed lignocellulosic biomass feedstock usually contains a mixture of pentoses (C5) such as xylose and arabinose, and hexoses (C6) such as glucose and mannose. Therefore, efficient utilization of C5 and C6 sugars is a prerequisite for a successful fermentation process with optimized carbon utilization. In this section, we review the studies focusing on clostridial mixed sugar fermentations producing butanol.

Mixed sugar fermentation studies date back to early 1980s, in which the researchers investigated the influence of different pentose and hexose sugars and their mixtures at different ratios on the fermentation kinetics [98]. Some clostridia have shown to readily consume sugar mixtures; however, they do so with poor efficiency [99]. Even though both strains can utilize glucose and xylose, *C. beijerinckii* has a large gene cluster containing most of the genes involved in xylose metabolism and regulation, while in *C. acetobutylicum* the xylose-related genes are dispersed over several different chromosomal locations [100]. Moreover, *C. beijerinckii* has more sets of xylose metabolic pathway genes than *C. acetobutylicum* [101]. Cells’ efficiency of simultaneously using sugars in mixed form decreases due to a phenomenon called carbon catabolite repression (CCR). Consequently, utilization of pentose sugars is reduced or prevented entirely in the presence of a preferred sugar such as glucose [94]. Furthermore, CCR can cause sequential utilization of sugars (diauxic growth) and a lag phase, which increases the residence time, thus operating costs. There have been attempts to improve product titers by using immobilized cultures [102], optimizing the culture pH and glucose to xylose ratio [103] and adding nutritional supplements [104] for fermentative butanol production from mixed sugars. In addition, genomic information [101, 105, 106] and transcriptome analysis results [107–112] of lignocellulosic sugar metabolisms and respective repression mechanisms are available in the literature.

There is ongoing research on metabolic engineering to develop clostridial strains capable of simultaneously fermenting hexose and pentose for butanol production [71, 93, 94, 113, 114]. Even though Lee et al. [23] stated metabolic engineering is necessary for simultaneous utilization of sugars, researchers have developed different feeding and pre-growth strategies achieving co-utilization without any strain manipulation [73, 115–120]. However, in the mixed sugar fermentation study of Zhang et al. [121], transcriptional studies suggested that glucose inhibition on xylose metabolism-related genes was still present despite the simultaneous utilization of glucose and xylose.

## Dataset development and exploratory data analysis for fermentative butanol production from lignocellulosic biomass and mixed sugars

By reviewing and extracting information from original research articles on clostridial fermentative batch production of butanol from lignocellulosic biomass and mixed sugars during the last three decades, we have developed a comprehensive dataset. 77 lignocellulosic hydrolysate, 19 lignocellulosic hydrolysate with additional glucose, and 79 mixed sugars fermentations have been included in the dataset, covering 175 fermentations in total. The dataset contains reported initial and final concentrations of all sugars and other components found in the substrates, all reported products in the fermentation broth, fermentation time, clostridial strain type, feedstock type, pretreatment method, and detoxification method used for those 175 fermentations. Latter four are summarized in Fig. 2 to illustrate their application frequencies. All the fermentations included in the dataset were conducted in batch mode. Quantification of fermentation products and substrates were done by using high pressure liquid chromatography and gas chromatography. As far as reported, data were directly derived from the article texts and tables, otherwise we used the WebPlotDigitizer tool [122] for mining the information from the plots. The dataset can be found in Additional file 1.

EDA is a statistical approach to analyze datasets for summarizing their main characteristics, which was promoted by John Tukey to encourage statisticians for in depth data exploration [123]. We used boxplot as a visual tool for EDA, which is a graphical method for illustration of numerical data groups through their quartiles that is the middle number between the smallest number and the median of the dataset. The lines extending vertically from the boxes called whiskers indicate the variability outside the upper and lower quartiles. In Figs. 4, 5, 6, boxplots are represented as rectangles with a vertical line showing the mean value, whiskers shown as dashed lines, and outliers are individual plus signs. We used built-in Matlab function to construct the boxplots.

22 fermentation variables from 175 fermentations were selected for EDA due to their importance for the process. Definitions and explanations of the fermentation variables can be found in “Substrate properties”, “Product mixture properties” and “Performance indicators” sections together with the results of EDA. It is important to note that the dataset could only include what was reported in the papers; there is therefore a possibility of unreported, unidentified and undetected components in the hydrolysates affecting production results and performance indicators.

### Substrate properties

**Fig. 3:**
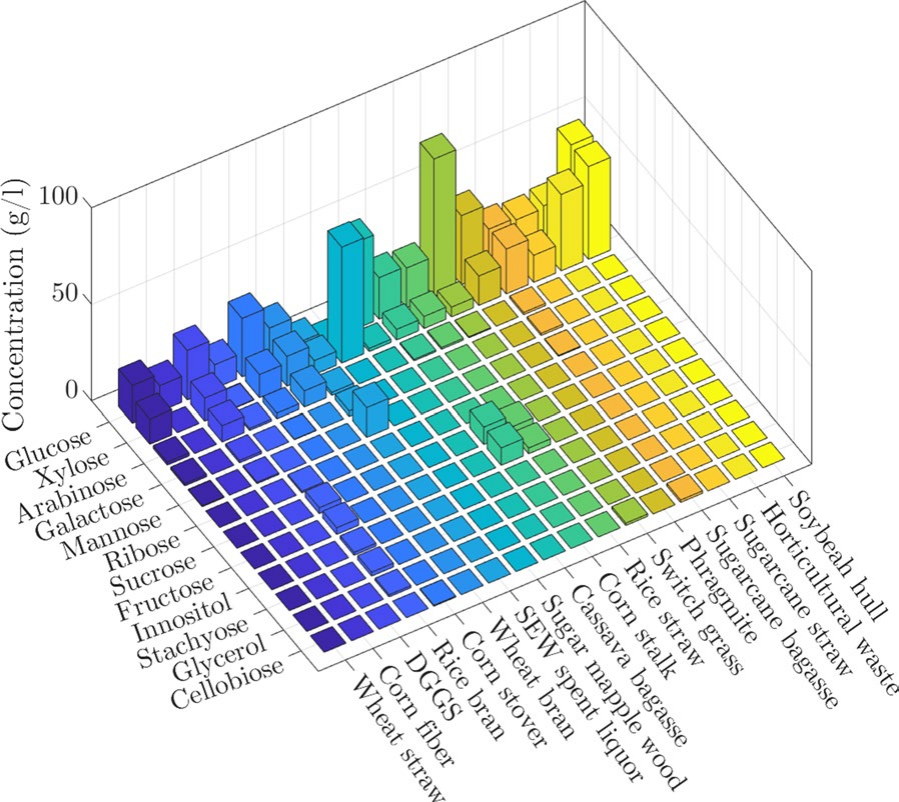
Concentrations of substrate components present in the lignocellulosic hydrolysates

In lignocellulosic substrate fermentation, the hydrolysate represents the sole source of carbon; however, microorganisms also need other nutrients such as nitrogen, phosphorous, sulfur, vitamins and minerals for growth and production. Typically, P2 stock solution and yeast extract are added externally, which increases the substrate cost [41–44, 48–73]. To tackle this problem, there have been attempts to provide the essential nutrients from waste materials such as wastewater sludge [55]. Optimization of medium components to minimize the substrate cost is important to consider when designing a fermentation process [18]. As discussed earlier, substrate composition has a great influence on fermentation; therefore, average amounts of 12 different components found in hydrolysates of 17 different lignocellulosic feedstock are shown in Fig. 3.

**Fig. 4:**
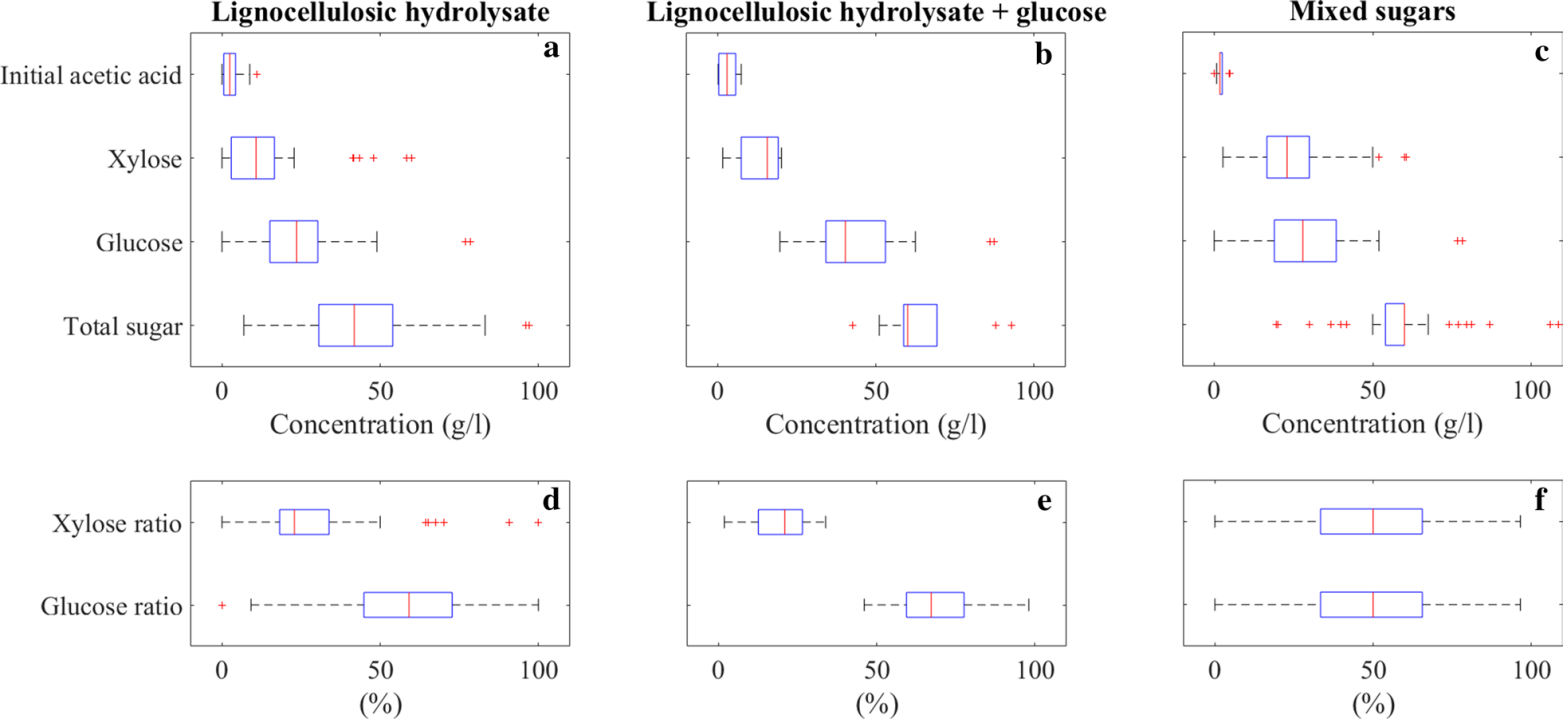
Substrate properties of lignocellulosic hydrolysate (**a**, **d**), lignocellulosic hydrolysate with additional glucose (**b**, **e**), and mixed sugar fermentations (**c**, **f**)

Figure 3 shows that glucose and xylose were the most commonly found sugar monomers in lignocellulosic hydrolysates. Therefore, initial concentrations (g/l) of these two sugars were selected as fermentation variables for EDA together with the total sugar concentration (g/l). We chose glucose ratio (glucose concentration/total sugar concentration × 100%) and xylose ratio (xylose concentration/total sugar concentration × 100%) as fermentation variables as well as initial acetic acid concentration (g/l) since it is often produced during pretreatment and has substantial effects on fermentation [101]. Thus, we identified 6 fermentation variables in total for EDA to investigate substrate properties as shown in Fig. 4.

In Fig. 4, initial concentrations of total substrates and their common constituents glucose, xylose, acetic acid as well as glucose and xylose ratios are shown for lignocellulosic hydrolysate, lignocellulosic hydrolysate with glucose, and mixed sugar fermentations.

For lignocellulosic hydrolysates, the medians of total sugars, glucose, xylose and initial acetic acid concentrations were 41.8, 23.6, 10.8 and 2.5 g/l, respectively. Outliers worth to mention include soybean hull hydrolysis yielding 49 g/l glucose and 48 g/l xylose [72], switchgrass yielding 77 g/l glucose with total sugar of 82 g/l [51], horticultural waste with 6 g/l glucose and 58 g/l xylose [71], and sugarcane bagasse containing 15 g/l glucose and 44 g/l xylose [42]. The deviation from the general trend could be due to the feedstock properties as well as the specific pretreatment methods.

Addition of glucose to the hydrolysate is a common practice to increase the total sugar concentration in the fermentation medium. Therefore, the total and individual sugar concentrations were higher for lignocellulosic hydrolysates with glucose. The medians of total sugar, glucose, and xylose concentrations were 60.05, 40.4, and 15.7, respectively. Glucose was occasionally added to wheat straw hydrolysate incrementally until the microorganisms were inhibited due to high substrate concentration [41], which resulted in outliers in Fig. 4b together with a case where glucose was added to cassava bagasse hydrolysate [60]. It is important to mention the change in glucose and xylose ratios due to addition of glucose. Xylose ratio decreased from 22.9 to 20.9%, while glucose ratio increased from 59 to 67%.

Mixed sugar fermentations are frequent among published lignocellulosic biomass fermentation studies. Researchers mimic the composition of hydrolysates with synthetic sugars to test the effect of impurities and inhibitors. Mixed sugar concentration values are more disperse with the medians of total sugar, glucose, and xylose concentrations of 60, 28, and 23 g/l, respectively. Interestingly, glucose and xylose ratios were both 50%. Even though the mixed sugar fermentation studies aim to mimic the original hydrolysate mixtures, their experimental conditions deviate from the actual values. This difference leads to the necessity for a holistic approach as in the objective of this critical review.

### Product mixture properties

**Fig. 5:**
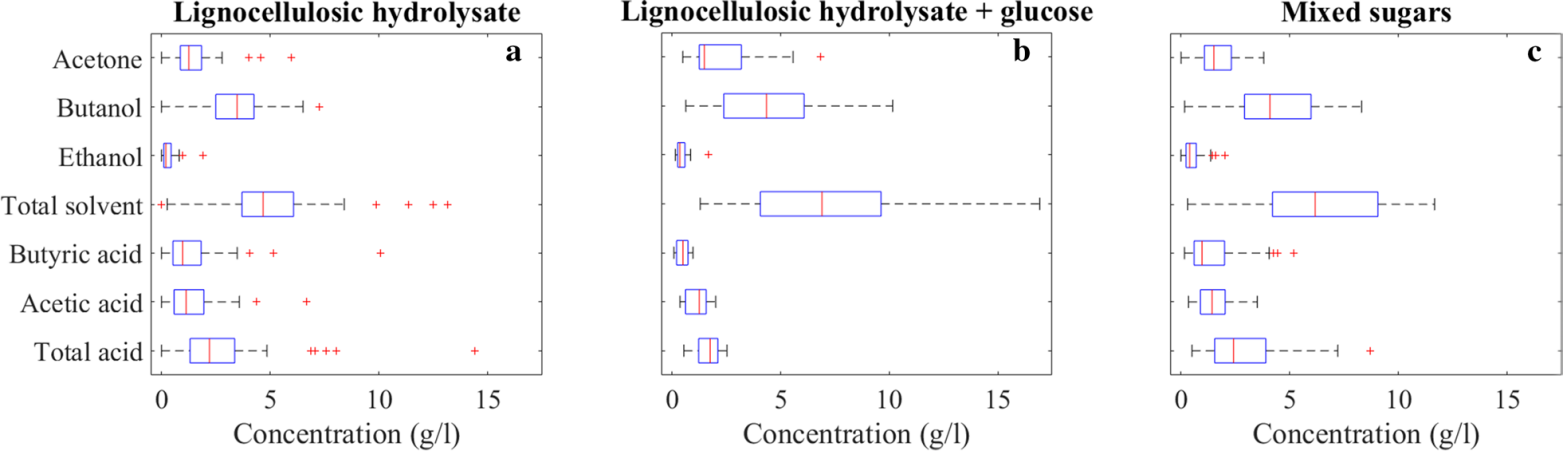
Product concentrations of lignocellulosic hydrolysate (**a**), lignocellulosic hydrolysate with additional glucose (**b**), and mixed sugar fermentations (**c**)

Maximization of butanol titer is an all-time objective as discussed above. Moreover, the product mixture can give an idea of the state of the fermentation. Therefore, we took a closer look at the composition and concentrations of the product mixtures, and identified 7 fermentation variables in total for EDA to investigate product mixture properties as shown in Fig. 5.

For lignocellulosic hydrolysate fermentations, the medians of total ABE solvents, acetone, butanol and ethanol concentrations are 9.33, 2.5, 6.95 and 0.4 g/l, respectively, while the medians of total acid, butyric acid and acetic acid concentrations are 4.4, 1.94 and 2.26 g/l. The highest reported value of our product of interest, butanol, was 14.5 g/l produced by *C. beijerinckii* P260 [58], shown as an outlier in Fig. 5b. High total acid concentrations of 14.1 and 15.1 g/l were reported for switchgrass hydrolysate fermentations by *C. acetobutylicum* 824, and those were reduced to 5.38 and 4.8 g/l after detoxification of the substrate with more than 100% increase in the total ABE solvent concentrations [51]. Total acid concentrations of 16.1 and 28.8 g/l were reported for soybean hull as the feedstock and engineered *C. tyrobutyricum* strains [72]. High acetone concentration in the product mixture is not desirable since it is corrosive to plastic piping and increases downstream costs. Therefore, wheat straw hydrolysate fermentation by *C. beijerinckii* with 11.9 g/l acetone [41] and switchgrass and phragmite hydrolysate fermentations by *C. saccharobutylicum* with 9.13 and 9.15 g/l acetone [65], respectively, are worth to mention.

For lignocellulosic hydrolysate with glucose fermentations, the medians of total ABE solvents, acetone, butanol and ethanol concentrations are 13.81, 2.97, 8.69 and 0.71 g/l, respectively, which are 48%, 19%, 25% and 78% higher than fermentations of lignocellulosic hydrolysates only as reported above. Medians of total acid, butyric acid and acetic acid concentrations are 3.5, 1.0 and 2.5 g/l, respectively. Additional glucose resulted in an increase in ABE solvents, and a decrease in total acids, indicating that the fermentations were closer to completion. Fermentation of wheat straw hydrolysate with added glucose by *C. beijerinckii* yielded a high acetone concentration of 13.7 g/l [39].

For mixed sugar fermentations, the medians of total ABE solvents, acetone, butanol and ethanol concentrations are 12.33, 3.01, 8.17 and 0.8 g/l, respectively, while those of total acid, butyric acid and acetic acid concentrations are 4.83, 1.93 and 2.85 g/l. Even though the initial total substrate concentrations of lignocellulosic hydrolysate with glucose and mixed sugar fermentations were almost the same, the latter had 12% lower ABE solvents, and 38% higher total acids. Reasons can be the difference in individual sugar concentrations and the stimulatory effects of compounds present in the hydrolysates [76].

### Performance indicators

**Fig. 6:**
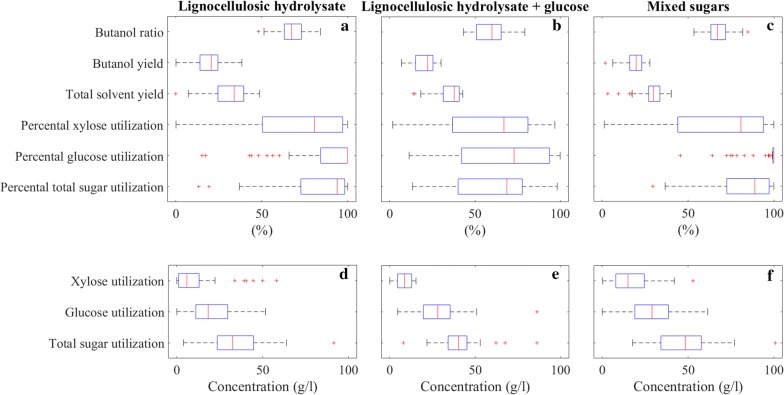
Performance indicators of lignocellulosic hydrolysate (**a**, **d**), lignocellulosic hydrolysate with additional glucose (**b**, **e**), and mixed sugar fermentations (**c**, **f**)

We identified percental (%) utilizations of total sugar, glucose, xylose and arabinose as common individual sugars, butanol and solvent yields in % (g product/g total sugar consumed × 100%) and the butanol ratio in % in ABE solvents (g butanol/g ABE solvents × 100%) as the performance indicators for a successful fermentation. Therefore, 9 fermentation variables were considered in total for EDA to investigate performance indicators as shown in Fig. 6. Even though solvent and/or butanol productivity is another important measure, reported values were difficult to compare due to the presence of lag phases and low data density, making it difficult to determine the exact time when fermentation had stopped.

For lignocellulosic hydrolysate fermentations, the medians of total sugar, glucose, and xylose utilizations (%) are 94, 100, and 80.8, respectively, which indicates a rather inefficient use of xylose. Lowest glucose utilizations shown as outliers in Fig. 6a are 49% for rice straw hydrolysate fermentation by non-acetone forming *C. sporogenes* [63] and 16% for switchgrass hydrolysate fermentation by *C. acetobutylicum* 824 that increased to 60% after detoxification [51]. In a similar manner, 14% glucose utilization during wheat straw hydrolysate fermentation by *C. beijerinckii* DSM 6422 increased to 76% after detoxification [52]. Solvent and butanol yields are important measures of cells’ efficiency to convert substrate to useful products, and a higher butanol ratio is desirable to minimize downstream processing costs. The medians of total ABE solvent yield, butanol yield and butanol ratio were 34%, 25.6% and 67.5%. The highest butanol yield with 38.4% was achieved for rice bran hydrolysate fermentation by *C. beijerinckii* NCIMB 8052 [56], which represents 94% of the maximum theoretical butanol yield from glucose, 0.41 (g/g) [124]. The highest butanol ratio in ABE solvents was 84.2% achieved in the same fermentation [56]. It is interesting to note that the butanol ratio was only 64% in the fermentation by non-acetone forming *C. sporogenes* [63], which can still be favorable, since the ethanol and butanol blend is already a valuable and useful product mix.

For lignocellulosic hydrolysate fermentations with added glucose, the medians of total sugar, glucose, and xylose utilizations were 68.7%, 73%, and 67%, respectively, which are lower than in fermentations of the hydrolysates without added glucose. The reason can be that the substrate concentrations reached inhibitory levels with the added glucose and consequently sugar utilizations became inefficient. Median values of total ABE solvent yield, butanol yield and butanol ratio were 37.8%, 22.3% and 60%. Despite 11.2% higher solvent yield, butanol yield and butanol ratio were 13% and 12.5% lower compared to lignocellulosic hydrolysate fermentations without extra glucose, which implies that the composition of the sugar mixture has an influence on the product mixture.

For mixed sugar fermentations, the medians of total sugar, glucose, and xylose utilizations are 89%, 100%, and 80.8%, respectively. Despite the similar initial total substrate concentrations of lignocellulosic hydrolysate with glucose and mixed sugar fermentations, the latter had 20% higher total sugar utilization. This can be due to the difference in concentrations of individual sugars and other medium components. Median values of total ABE solvent yield, butanol yield and butanol ratio were 29.9%, 19.8% and 67%. Both ABE solvent and butanol yield values were significantly lower than in lignocellulosic hydrolysate with added glucose fermentations. However, the butanol ratio was 11.7% higher in mixed sugar fermentations.

### Correlations between fermentation variables

**Fig. 7:**
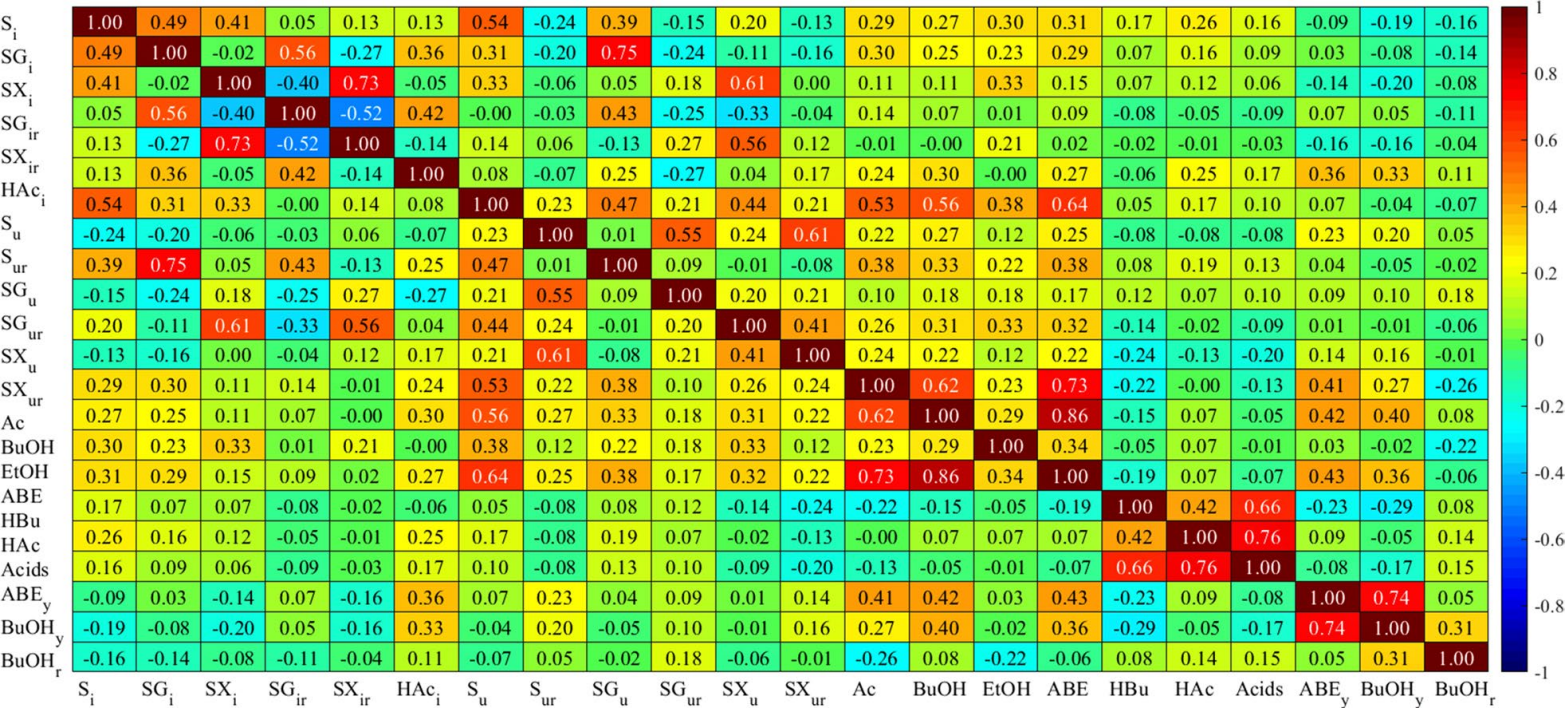
Correlation coefficients between all 22 fermentation variables

We chose Kendall’s correlation coefficient to determine the correlations between variables since it is able predict nonlinear relationships [125] and robust in presence of outliers in data [126]. The coefficient has a value between + 1 and − 1, where 1 is total positive correlation, 0 is no correlation, and − 1 is total negative correlation. All 22 fermentation variables introduced in the previous section was used. They are initial substrate, glucose, xylose and acetic acid concentrations (S_i_, SG_i_, SX_i_ and HAc_i_), ratio of glucose and xylose in the initial substrate mixture (SG_ir_ and SX_ir_), utilized concentrations of total substrate, glucose and xylose (S_u_, SG_u_ and SX_u_), percental utilizations of total substrate, glucose and xylose (S_ur_, SG_ur_ and SX_ur_), concentrations of acetone, butanol, ethanol, ABE solvents, butyric acid, acetic acid and total acids (Ac, BuOH, EtOH, ABE, HBu, HAc and acids), ABE solvents and butanol yields (ABE_y_ and BuOH_y_), and butanol ratio in ABE solvents (BuOH_r_). Figure 7 shows values of Kendall’s correlation coefficients for each pair of 22 fermentation variables.

All utilized sugar concentrations (S_u_, SG_u_, SX_u_) increase as their initial concentrations (S_i_, SG_i_, SX_i_) increase, which reflects into positive and statistically significant correlation coefficients. On the other hand, sugar utilizations (S_ur_, SG_ur_, SX_ur_) (%) decrease with increasing initial total sugar (S_i_) and glucose concentrations (SG_i_). Even though higher sugar concentration improves fermentation to some extent, beyond some threshold, it starts to become inhibitory and this phenomenon is illustrated with negative correlation coefficients. Furthermore, correlation coefficients show that SG_ur_ decreases with increasing initial glucose ratio (SG_ir_) with correlation coefficient value of − 0.25 and increases with increasing initial xylose ratio (SX_ir_) in the substrate with correlation coefficient value of 0.27. This seems controversial at first sight. However, high SG_ir_ is a result of high SG_i_, which leads to lower glucose utilization as explained above. CCR information is another important feature extracted from the correlations. A higher initial glucose ratio (SG_ir_) and a lower initial xylose ratio (SX_ir_) leads to an increasing utilized glucose concentration (SG_u_) with a correlation coefficient of − 0.13 for the latter. Similarly, the utilized xylose concentration (SX_u_) increases with an increasing initial xylose ratio (SX_ir_), while it decreases as the initial glucose ratio (SG_ir_) increases with a correlation coefficient of − 0.33. Therefore, both sugars repress each other’s utilization due to CCR. However, the repression effect is greater from glucose to xylose (|− 0.33| > |− 0.13|) as suggested in our previous work cite [127].

As the correlation coefficients in Fig. 7 indicate, all product concentrations increase with increasing initial concentrations of all sugars; acetone (Ac) and butanol (BuOH) concentrations are more influenced by initial glucose (SG_i_) than xylose (SX_i_), and SGi has a greater influence on Ac (0.30) than on BuOH (0.25). This is in line with previous work where no acetone accumulation was found during fermentation of xylose by *C. acetobutylicum* [109]. Both solvent yield (ABE_y_) and butanol yield (BuOH_y_) decrease with increasing initial xylose concentration (SX_i_) and ratio (SX_ir_), while they increase with elevated initial acetic acid concentration (HAc_i_) that is often generated during pretreatment of lignocellulosic biomass. Negative correlation coefficient between xylose and yields could be due to the carbon content of one xylose molecule containing one carbon less than glucose, thus one xylose molecule has less capacity to yield products. Positive correlation between ABE_y_ and BuOH_y_, and HAc_i_ can be due to presence of acetic acid in the beginning of fermentation facilitating solvent formation, which is line with metabolic pathway of fermentation [6]. Another crucial performance indicator, the butanol ratio (BuOH_r_), becomes greater as S_i_, SG_i_, and SG_ir_ decrease. Furthermore, all product concentrations except HBu and EtOH increase with increasing HAc_i_. Some researchers stated that high initial acetic acid concentrations could facilitate acetone formation, consequently increase the acetone to butanol ratio [101]. However, the correlation coefficient between HAc_i_ and BuOH is greater than that of HAc_i_ and Ac, i.e. 0.30 and 0.24, respectively. Therefore, a potential effect of initial acetic acid concentrations in the fermentation medium on product formation needs to be studied in more detail.

Correlation coefficients between utilized sugar concentrations (S_u_, SX_u_) and sugar utilizations (S_ur_, SX_ur_) are positive, indicating the more the utilized sugar concentration, the higher the utilization (%) with respect to its initial concentration. In addition, all solvent concentrations (ABE, Ac, BuOH, EtOH) increase with increasing S_u_, SG_u_, SX_u_, S_ur_, SG_ur_, and SX_ur_. One exception to this trend is that there is no significant correlation between SG_ur_ and Ac. HAc is in positive correlation with S_u_ and SG_u_, while HBu is in negative correlation with SX_u_ and SX_ur_. Even though both acids are produced as the cells metabolize glucose and xylose, the difference in the effects of specific sugars in the metabolic pathway is apparent.

Ac and BuOH concentrations decrease with increasing HBu concentration, while there is no correlation with HAc. Therefore, the HBu concentration alone can be considered as a measure of fermentation completion. EtOH is not correlated with any of the acid products, which is in good agreement with the kinetic model developed by Shinto et al. [128].

## Conclusions

To develop new strategies to increase the overall competitiveness of fermentative butanol production from lignocellulosic biomass by clostridia, it is crucial to have an overall view of all relevant process aspects and characteristics. One of the main challenges is expensive fermentation substrate, accounting for 66% of the total costs [33]. Besides availability, supply and storage issues, efficient conversion of the feedstock to fermentable sugars remain costly. Therefore, it is significant to develop new, more cost-efficient pretreatment methods that minimize the generation of inhibitors, energy consumption, operating cost, and simultaneously maximize fermentable sugar yield with a careful consideration of feedstock properties. In addition, adapting and developing strains, which can effectively utilize all the sugars present in the substrate [56] and better tolerate inhibitors can contribute to the solution. Downstream processing has the second highest share in the overall cost (16%) [33]. Higher product concentrations and ratios can make this process more economically feasible. However, the solvent toxicity problem and the generation of undesirable by-products, i.e. acetone and ethanol, are necessary to overcome. Even though metabolic engineering has provided different alternatives such as improved solvent tolerance and non-acetone forming strains, those still need to be tested over a prolonged time under different operating conditions and further improved e.g. by means of systems biology guided strain engineering and Synthetic biology to gain and sustain industrial viability.

Understanding the process variables and performance indicators has been lacking to a great extent, since many studies narrow their focus to a particular problem and evaluate their solution in the same narrow window. To illustrate the benefits of having a holistic view, we have developed a dataset by collecting fermentation data and performed an EDA. The results show that common practices such as addition of glucose to achieve a high sugar concentration could have detrimental effects on production performance, and unexpected trends might occur depending on process design choices. The EDA results provided insight into typical operating conditions as well as performance indicators. The correlation results showed that common practices such as addition of glucose to achieve a high sugar concentration could have detrimental effects on production performance. Moreover, correlation between different fermentation variables revealed several important trends, which confirms previous observations or contradicts some current argumentations. Correlation between the initial ratios of glucose and xylose and their utilizations during mixed sugar fermentations unveiled that CCR was active for both sugars, confirming previous observations [127]. On the other hand, our correlation findings contradict with the common belief that the acetic acid generated in biomass pretreatment can result in increased acetone formation. Therefore, it is very important to note that correlation does not necessarily imply causation and a careful attendance is necessary when utilizing correlation information in design of processes both in lab scale and in industrial scale. Furthermore, usefulness of EDA results heavily depends on the quality of the reported data. Further efforts in the field need to focus on reporting detailed information about pretreatment conditions and studying their influence on fermentation performance. All in all, this approach provides different insights and information as a guide to a more successful fermentation of lignocellulosic sugars to butanol as a key to more competitive butanol production processes for biofuel applications in the future.

## Additional file

**Additional file 1.** Fermentation dataset.

## Data Availability

All data generated or analyzed during this study are included in this published article and its additional file.
